# Human brain activity and functional connectivity associated with verbal long-term memory consolidation across 1 month

**DOI:** 10.3389/fnhum.2024.1342552

**Published:** 2024-02-21

**Authors:** Catherine W. Tallman, Zhishang Luo, Christine N. Smith

**Affiliations:** ^1^Department of Psychology, University of California, San Diego, San Diego, CA, United States; ^2^Veterans Affairs San Diego Healthcare System, Department of Research Service, San Diego, CA, United States; ^3^Halıcıoğlu Data Science Institute, University of California, San Diego, San Diego, CA, United States; ^4^Department of Psychiatry, University of California, San Diego, San Diego, CA, United States; ^5^Center for the Neurobiology of Learning and Memory, University of California, Irvine, Irvine, CA, United States

**Keywords:** memory consolidation, verbal memory, functional magnetic resonance imaging (fMRI), functional connectivity, hippocampus, systems consolidation

## Abstract

**Introduction:**

Declarative memories are initially dependent on the hippocampus and become stabilized through the neural reorganization of connections between the medial temporal lobe and neocortex. The exact time-course of these neural changes is not well established, although time-dependent changes in retrieval-related brain function can be detected across relatively short time periods in humans (e.g., hours to months).

**Methods:**

In a study involving older adults with normal cognition (N = 24), we investigated changes in brain activity and functional connectivity associated with the long-term memory consolidation of verbal material over one month. Participants studied fact-like, three-word sentences at 1-month, 1-week, 1-day, and 1-hour intervals before a recognition memory test inside an MRI scanner. Old/new recognition with confidence ratings and response times were recorded. We examined whole-brain changes in retrieval-related brain activity, as well as functional connectivity of the hippocampus and ventromedial prefrontal cortex (vmPFC), as memories aged from 1 hour to 1 month. Secondary analyses minimized the effect of confounding factors affected by memory age (i.e., changes in confidence and response time or re-encoding of targets).

**Results:**

Memory accuracy, confidence ratings, and response times changed with memory age. A memory age network was identified where retrieval-related brain activity in cortical regions increased or decreased as a function of memory age. Hippocampal brain activity in an anatomical region of interest decreased with memory age. Importantly, these changes in retrieval-related activity were not confounded with changes in activity related to concomitant changes in behavior or encoding. Exploratory analyses of vmPFC functional connectivity as a function of memory age revealed increased connectivity with the posterior parietal cortex, as well as with the vmPFC itself. In contrast, hippocampal functional connectivity with the vmPFC and orbitofrontal cortex decreased with memory age.

**Discussion:**

The observed changes in retrieval-related brain activity and functional connectivity align with the predictions of standard systems consolidation theory. These results suggest that processes consistent with long-term memory consolidation can be identified over short time periods using fMRI, particularly for verbal material.

## 1 Introduction

Long-term memory consolidation is the time-dependent neural reorganization that establishes enduring memories from unstable memory traces. According to standard systems consolidation theory (SCT), as time passes after learning, declarative memories that were initially dependent on the hippocampus become stabilized in the neocortex and can eventually be retrieved independently of the hippocampus (Marr, 1971; McClelland et al., 1992, 1995; Alvarez et al., 1994). Patients with lesions restricted to the hippocampus demonstrated that memories several years old can be retrieved independent of the hippocampus, and this pattern of temporally graded retrograde amnesia extended further when the parahippocampal gyrus was also damaged (Manns et al., 2003; Bayley et al., 2006). However, competing theories of memory consolidation such as multiple-trace/transformation theory (MTT/TT) (Nadel and Moscovitch, 1997; Moscovitch et al., 2005; Sekeres et al., 2017) and contextual binding theory (CBT) (Yonelinas et al., 2019) debate the dependence on the hippocampus for remote episodic memory retrieval. Specifically, these theories posit that the hippocampus is always necessary for memory retrieval so long as the memories retain a sufficient level of detail and/or associative information about the episodic event (Sekeres et al., 2017; Yonelinas et al., 2019). The theories described above are in agreement that semantic memories (memories that have lost information about the context in which they were learned) become hippocampus independent over time.

Although the predictions of long-term memory consolidation theories consider whether or not the hippocampus is *necessary* for remote memory retrieval, non-invasive imaging techniques such as functional magnetic resonance imaging (fMRI) allow for the examination of relative changes in retrieval-related brain function over time. Time-dependent decreases in MTL brain activity associated with semantic (“fact”) memory retrieval across several years demonstrated changes in brain function consistent with SCT (Haist et al., 2001; Douville et al., 2005; Smith and Squire, 2009). Studies of time-dependent changes associated with autobiographical memory retrieval are less consistent as some studies demonstrated decreases in hippocampal activity (e.g., Maguire and Frith, 2003; Gilboa et al., 2004) or increases in hippocampal activity (e.g., Piolino et al., 2004; Rekkas and Constable, 2005) across several years. Overall, the examination of changes in retrieval-related brain activity in humans across years reveals that decreasing activity can be detected over this time period.

In studies of rodents and primates with hippocampal lesions, the time-dependent changes of systems consolidation appears to be shorter, with memories becoming hippocampus independent over days to weeks (Winocur, 1990; Zola-Morgan and Squire, 1990; Kim and Fanselow, 1992). Studies of hippocampal activity in rodents across short time periods using markers of brain activity such as glucose metabolism or immediate early gene activity also reveal evidence that hippocampal activity decreases with memory age (Bontempi et al., 1999; Frankland et al., 2004; Maviel et al., 2004; Wheeler et al., 2013). Nevertheless, there are reports of hippocampal activity in rodents that increased for recent and remote time points or that was similar for these time points (Teixeira et al., 2006; Makino et al., 2019), suggesting that patterns of hippocampal findings are not entirely consistent within the animal literature.

In concordance with the animal literature, studies of human neuroimaging and consolidation have also tried to detect changes in hippocampal activity over short time periods (minutes to months). Several studies observed hippocampal activity decreases with memory age, supporting the predictions of systems consolidation theory (left hippocampus: Bosshardt et al., 2005b; Takashima et al., 2006, 2009; Sterpenich et al., 2009; Yamashita et al., 2009; Smith et al., 2010; Milton et al., 2011; Furman et al., 2012; Harand et al., 2012; Ritchey et al., 2015; Dandolo and Schwabe, 2018; Sekeres et al., 2018; associative memory: Du et al., 2019). Decreasing activity could also be taken as support for TT and CBT due to forgetting of memory details/associations in the remote conditions. Yet, most of these studies queried associative memory and still found decreases in activity in memory age. Other studies found increases in hippocampal activation associated with memory age (Bosshardt et al., 2005a; right hippocampus: Bosshardt et al., 2005b; Gais et al., 2007; Smith et al., 2010; Vanasse et al., 2022), which can be taken as support for the idea that memory traces increase within the MTL as time passes after learning, as predicted by MTT. Additionally, several studies have found no change in retrieval-related hippocampal activity associated with memory age at varying time intervals up to ∼45 days (Stark and Squire, 2000; Janzen et al., 2008; Suchan et al., 2008; Davis et al., 2009; Vilberg and Davachi, 2013; Tompary and Davachi, 2017; item memory: Du et al., 2019; Tallman et al., 2022b). Note many of these “null” effects could be taken as support for MTT, TT, or CBT because these studies tested associative memory. Null effects can also reflect low statistical power to detect time-dependent changes in hippocampal activity (see Tallman et al., 2022a for further discussion of null effects).

There are several methodological factors to consider (e.g., experimental design, time interval between recent and remote timepoints) which may explain these inconsistent findings across studies. In particular, it is unclear if the type of memoranda used is relevant to discern a common pattern of hippocampal activity changes. Several studies have examined memory for verbal material, and the pattern of hippocampal activity changes remains variable. Some studies identified decreases in hippocampal activity with memory (left hippocampus: Bosshardt et al., 2005b; Ritchey et al., 2015; associative memory: Du et al., 2019), others identified increases (Bosshardt et al., 2005a; right hippocampus: Bosshardt et al., 2005b; Gais et al., 2007), and others failed to detect any changes in activity (Davis et al., 2009; item memory: Du et al., 2019).

Theories of long-term memory consolidation are dependent on changing hippocampal-cortical and cortico-cortical *connections*. Thus, assessing time-dependent changes in functional connectivity, rather than overall differences in brain activity, may be a more reliable method for detecting long-term memory consolidation effects. Changes in hippocampal functional connectivity of semantic memory have not yet been examined, although studies of autobiographical memory exhibited decreasing hippocampal-cortical functional connectivity across several years or more (Söderlund et al., 2012; Sheldon and Levine, 2013; Gilmore et al., 2021). Time-dependent decreases in hippocampal-cortical functional connectivity were also detected over a short time period of 1 day in humans (Takashima et al., 2009; Brodt et al., 2016) and similarly across days in rodents (Bontempi et al., 1999; Wheeler et al., 2013; Wirt and Hyman, 2019). Assessing more direct changes in the relationship between the MTL and cortex with functional connectivity may reveal more consistent patterns of brain function associated with long-term memory consolidation, particularly over short time periods.

Therefore, we examined brain activity and functional connectivity in older adults associated with retrieval of unique sets of three-word sentences studied 1 hour, 1 day, 1 week, or 1 month (four memory ages) before a memory retrieval test. Older adults were tested because this group of participants also completed a companion study (manuscript in preparation), which examined news event memory for the recent and remote past across decades, a task that necessitates examining memory in older adults. Our aim was to identify time-dependent patterns of retrieval-related brain activity and functional connectivity associated with memory age. Secondary analyses were conducted to reduce the impact of additional factors that changed with memory age (i.e., changes in confidence and response time, re-encoding of targets) and determine if they influenced the primary analyses.

## 2 Materials and methods

### 2.1 Participants

Twenty-eight participants (12 female; mean age = 72.6 years ± 1.5 years; range = 65–91 years; mean education = 16.6 ± 0.4 years) were recruited from the San Diego community and underwent MRI scanning. One participant was excluded due to technical issues while MRI scanning, one participant was excluded due to excessive motion during scanning, and two participants were excluded due to difficulty understanding the task instructions. Twenty-four participants (10 female; mean age = 72.6 ± 1.3 years; range = 65–91 years; mean education = 16.6 ± 0.5 years) were included in the reported statistical analyses.

### 2.2 Study design

**FIGURE 1:**
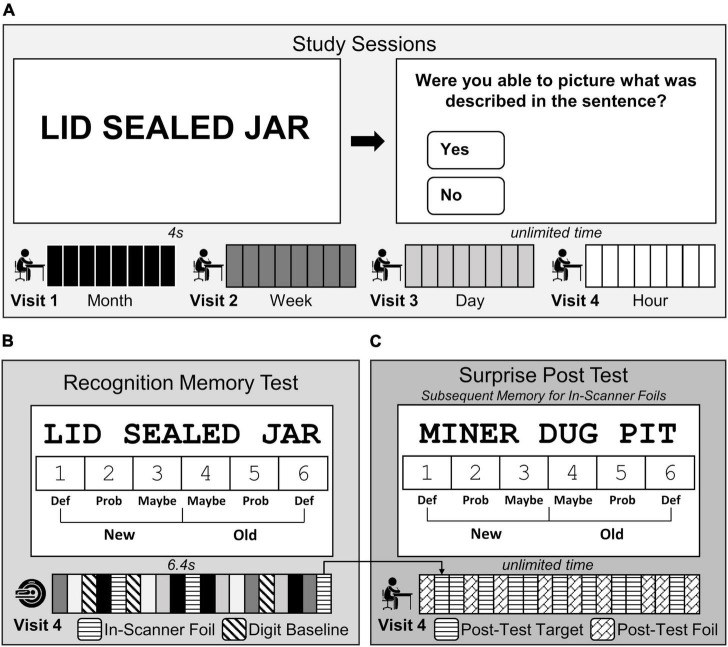
Three-word sentences task design. **(A)** Participants studied a unique set of fact-like, three-word sentences 1 month (black, Visit 1), 1 week (dark grey, Visit 2), 1 day (light grey, Visit 3), and 1 hour (white, Visit 4) before the MRI scanning session. **(B)** During Visit 4, participants completed a test in the scanner where they made old/new recognition memory judgments using confidence ratings (1 = definitely new to 6 = definitely old) in response to each three-word sentence studied previously (240 studied sentences; white, light grey, dark grey, and black bars) intermixed with 120 novel sentences (in-scanner foil; horizontal striped bars), and made even/odd judgments in response to digits (baseline trials; diagonally striped bars). **(C)** At the end of Visit 4, participants completed a surprise post-test outside of the scanner to examine subsequent memory for the in-scanner foils. Participants made memory judgments using the same confidence scale in **(B)**, but in response to the 120 post-test targets (previously presented in-scanner foils, horizontal striped bars) intermixed with a completely novel set of 120 sentences (post-test foils, diagonal brick bars).

Each participant completed the study protocol which consisted of (1) four study sessions outside of the scanner, (2) one recognition memory test while undergoing fMRI scanning, and (3) one surprise post-test outside of the scanner. Study sessions were administered online in the laboratory or at the participants’ home using Qualtrics software (Qualtrics, Provo, UT). They occurred at 1 month, 1 week, 1 day, and 1 hour before an in-scanner recognition memory test (Figure 1A; Visits 1–4). Participants learned a unique set of 60 fact-like, three-word sentences (e.g., LID SEALED JAR) at each of the four study sessions. Sentences were created such that no words were repeated across the stimuli. Each sentence was individually presented on the screen for 4 s followed by a question that encouraged deep, elaborative encoding (Could you picture what was described in the sentence?) with unlimited time to respond. To aid memory retention, the set of sentences was repeated 4 times at the study session.

Participants completed a recognition memory test of the previously studied sentences while undergoing fMRI scanning (Figure 1B; Visit 4). Within the scanning session, 240 three-word target sentences (60 from each study session), 120 novel three-word sentences, and 282 digit baseline trials were presented. Each three-word sentence trial was presented for 6.4 sec during which participants made a recognition memory judgment using confidence ratings (1 = definitely new, 2 = probably new, 3 = maybe new, 4 = maybe old, 5 = probably old, and 6 = definitely old). Confidence ratings were completed by selecting a number (1–6) on the screen with an MRI-compatible mouse (Current Designs, Philadelphia, PA, USA). The starting location of the mouse cursor on the screen was randomized before each sentence trial with the intention of decorrelating rightward and leftward movements with the right and left sides of the 1–6 scale. After each sentence trial, zero to seven digit baseline trials were presented (mean = 1.95 trials). For each baseline trial, a single digit (1–8) was presented and participants selected with the mouse whether it was even or odd (3.2 s) (Stark and Squire, 2001). The scanning session consisted of six 8.8 min runs, with each run containing 60 sentences [40 target sentences (10 from each memory age condition) intermixed with 20 novel foil sentences].

A surprise post-test to assess subsequent memory of in-scanner foils was administered immediately following the fMRI scan (Figure 1C). Participants were presented with the 120 foil three-word sentences viewed in the scanner (now considered to be targets) intermixed with 60 novel foil three-word sentences across 6 runs. The same recognition judgment scale was used, and participants had unlimited time to indicate if the sentence was old (previously seen in the scanner), or new (first time encountering the sentence in the study). The presentation of sentences was counterbalanced across participants so that each set of sentences had an equal chance of being presented during one of the study phases, during the test phase as a target or foil, or during the post-scan recognition memory test as a foil.

### 2.3 fMRI imaging protocol

Scanning was conducted on a 3T General Electric MR750 Discovery MRI scanner at the Center for Functional MRI (University of California, San Diego) using a NOVA 32 channel head coil. Functional images were acquired using a gradient- echo, echo-planar, T2*-weighted pulse sequence, using parameters closely matched to the HCP Lifespan brain imaging protocol (800 msec TR; 37.0 msec TE; 104 × 104 matrix size; 20.8 cm field of view; 2 mm × 2 mm in-plane resolution; multiband acceleration factor = 8). Seventy-two axial slices (slice thickness = 2 mm) were acquired covering the whole brain. Spin-echo fieldmap scans with opposite phase encoding directions (*P* > *A* and *A* > *P*) were acquired for susceptibility induced distortion correction after run 1 to correct functional runs 1–3 and after run 4 to correct functional runs 4–6. Following the third functional run, high-resolution structural images were acquired using a sagittal T1-weighted MPRAGE pulse sequence (25.6 cm field of view; 160 slices; 1 mm slice thickness; 256 × 256 matrix size). PROMO (PROspective MOtion correction; White et al., 2009) was used to adaptively compensate for motion during structural scanning resulting in no loss of anatomical data due to subject motion.

### 2.4 Data analysis

#### 2.4.1 Behavioral data analysis

Measures of discrimination, confidence, and response time for each memory age condition were calculated by taking the mean across all targets. Discriminability [d prime (*d’*)] was also calculated using the following formula: Z (hit rate)–Z (false alarm rate) using Excel. Means and SEM are reported. Significant changes across memory age conditions were tested using repeated measures ANOVA.

#### 2.4.2 Neural data pre-processing

Results included in this manuscript come from pre-processing performed using fMRIPrep 21.0.2 (Esteban et al., 2018a,b; RRID:SCR_016216) which is based on Nipype 1.6.1 (Gorgolewski et al., 2011; RRID:SCR_002502; Gorgolewski et al., 2018). Additional pre-processing and statistical modeling for the neuroimaging analyses was conducted using programs from Analysis of Functional NeuroImages (AFNI) (Cox, 1996).

##### 2.4.2.1 fMRIprep anatomical data pre-processing

One T1-weighted (T1w) image was corrected for intensity non-uniformity (INU) with N4BiasFieldCorrection (Tustison et al., 2010), distributed with ANTs 2.3.3 (Avants et al., 2008, RRID:SCR_004757), and used as T1w-reference throughout the workflow. The T1w-reference was then skull-stripped with a Nipype implementation of the antsBrainExtraction.sh workflow (from ANTs), using OASIS30ANTs as target template. Brain tissue segmentation of cerebrospinal fluid (CSF), white-matter (WM) and gray-matter (GM) was performed on the brain-extracted T1w using fast (FSL 6.0.5.1:57b01774, RRID:SCR_002823, Zhang et al., 2001) Brain surfaces were reconstructed using recon-all (FreeSurfer 6.0.1, RRID:SCR_001847, Dale et al., 1999), and the brain mask estimated previously was refined with a custom variation of the method to reconcile ANTs-derived and FreeSurfer-derived segmentations of the cortical gray-matter of Mindboggle (RRID:SCR_002438, Klein et al., 2017). Volume-based spatial normalization to one standard space (MNI152NLin2009cAsym) was performed through non-linear registration with antsRegistration (ANTs 2.3.3), using brain-extracted versions of both T1w reference and the T1w template. The following template was selected for spatial normalization: ICBM 152 Non-linear Asymmetrical template version 2009c [(Fonov et al., 2009), RRID:SCR_008796; TemplateFlow ID: MNI152NLin2009cAsym].

##### 2.4.2.2 fMRIprep functional data pre-processing

For each of the 6 BOLD runs, the following pre-processing was performed. First, a reference volume and its skull-stripped version were generated using a custom methodology of fMRIPrep. Head-motion parameters with respect to the BOLD reference (transformation matrices, and six corresponding rotation and translation parameters) are estimated before any spatiotemporal filtering using mcflirt (FSL 6.0.5.1:57b01774, Jenkinson et al., 2002). The BOLD time-series (including slice-timing correction when applied) were resampled onto their original, native space by applying the transforms to correct for head-motion. These resampled BOLD time-series will be referred to as pre-processed BOLD in original space, or just pre-processed BOLD. The BOLD reference was then co-registered to the T1w reference using bbregister (FreeSurfer) which implements boundary-based registration (Greve and Fischl, 2009). Co-registration was configured with six degrees of freedom. Several confounding time-series were calculated based on the pre-processed BOLD: framewise displacement (FD), DVARS and three region-wise global signals. FD was computed using two formulations following Power (absolute sum of relative motions, Power et al., 2014) and Jenkinson (relative root mean square displacement between affines, Jenkinson et al., 2002). FD and DVARS are calculated for each functional run, both using their implementations in Nipype (following the definitions by Power et al., 2014). The three global signals are extracted within the CSF, the WM, and the whole-brain masks. Additionally, a set of physiological regressors were extracted to allow for component-based noise correction (CompCor, Behzadi et al., 2007). Principal components are estimated after high-pass filtering the pre-processed BOLD time-series (using a discrete cosine filter with 128s cut-off) for the two CompCor variants: temporal (tCompCor) and anatomical (aCompCor). For aCompCor, three probabilistic masks (CSF, WM and combined CSF+WM) are generated in anatomical space. The implementation differs from that of Behzadi et al. (2007) in that instead of eroding the masks by 2 pixels on BOLD space, the aCompCor masks are subtracted a mask of pixels that likely contain a volume fraction of GM. This mask is obtained by dilating a GM mask extracted from the FreeSurfer’s aseg segmentation, and it ensures components are not extracted from voxels containing a minimal fraction of GM. Finally, these masks are resampled into BOLD space and binarized by thresholding at 0.99 (as in the original implementation). Components are also calculated separately within the WM and CSF masks. For each CompCor decomposition, the k components with the largest singular values are retained, such that the retained components’ time series are sufficient to explain 50 percent of variance across the nuisance mask (CSF, WM, combined, or temporal). The remaining components are dropped from consideration. The head-motion estimates calculated in the correction step were also placed within the corresponding confounds file. The confound time series derived from head motion estimates and global signals were expanded with the inclusion of temporal derivatives and quadratic terms for each (Satterthwaite et al., 2013). Frames that exceeded a threshold of 0.5 mm FD or 1.5 standardized DVARS were annotated as motion outliers. The BOLD time-series were resampled into standard space, generating a pre-processed BOLD run in MNI152NLin2009cAsym space. First, a reference volume and its skull-stripped version were generated using a custom methodology of fMRIPrep. All re-samplings can be performed with a single interpolation step by composing all the pertinent transformations (i.e., head-motion transform matrices, susceptibility distortion correction when available, and co-registrations to anatomical and output spaces). Gridded (volumetric) re-samplings were performed using antsApplyTransforms (ANTs), configured with Lanczos interpolation to minimize the smoothing effects of other kernels (Lanczos, 1964). Non-gridded (surface) re-samplings were performed using mri_vol2surf (FreeSurfer).

Many internal operations of fMRIPrep use Nilearn 0.8.1 (Abraham et al., 2014, RRID:SCR_001362), mostly within the functional processing workflow. For more details of the pipeline, see the section corresponding to workflows in fMRIPrep’s documentation.

##### 2.4.2.3 Additional functional data pre-processing

After pre-processing using fMRIprep, each functional run was subsequently smoothed up to a kernel of 4 mm (*3dBlurtoFWHMx)* and scaled so the mean activation for each voxel was 100. Timepoints identified as motion outliers by fMRIprep criteria were censored during subsequent statistical analysis (Power et al., 2014). As mentioned above, one participant was excluded as more than 10% of their functional data were identified as motion outliers. The remaining subjects included in the statistical analyses (*n* = 24) had on average 1.1% of their functional data censored due to motion artifacts.

#### 2.4.3 General linear modeling

Multiple regression analyses were conducted using AFNI’s *3dDeconvolve* to calculate beta coefficients for vectors of interest relevant to each analysis (described below). Confound regressors included in multiple regression analyses were selected using the aCompCor pipeline approach to optimize the removal of noisy signal when conducting task-related functional connectivity analyses (Mascali et al., 2021) and using fMRIprep. These regressors were the first 5 aCompCor components (principal components which capture anatomical noise) and 24 motion regressors (6 translation/rotation directions, 6 derivatives of translation/rotation directions, 6 squared translation/rotation directions, and 6 squared derivatives of translation/rotation directions).

The hemodynamic response function was independently modeled for each sentence trial using twenty-one TENT functions spanning 0–16 s after the onset of the sentences. Peak activation from 6.4–9.6 s post-stimulus onset was isolated for analysis. The deconvolution matrix was fit using generalized least square regression within *3dREMLfit* to reduce the influence of temporal autocorrelation. The resulting β coefficients for each vector of interest from the multiple regression model reflected the mean peak activation.

#### 2.4.4 Region of interest analysis: hippocampus

We defined an anatomical hippocampal region of interest (ROI) for each participant to examine brain activity changes associated with memory age as well as to use as a seed in the task-based functional connectivity analyses (described below). Bilateral hippocampal ROIs were created using FSL FIRST to segment each participant’s T1-weighted image co-registered to MNI space (Patenaude et al., 2011). The anterior and posterior hippocampus was defined by the coronal MRI slice where the uncus was no longer visible, which demarcated the beginning of the posterior hippocampal ROI.

#### 2.4.5 Whole-brain voxel-wise analysis of brain activity

##### 2.4.5.1 Primary analysis: memory age

The purpose of this study was to identify retrieval-related brain activity that changed as a function of memory age and that was consistent with memory consolidation. Accordingly, we were interested in brain activity that increased or decreased in relatively monotonic *patterns* across the memory age conditions. The detection of a pattern of activity across several memory ages is likely more robust than detection of differential activity across only two memory ages. Therefore, we *a priori* tested for retrieval-related brain regions which demonstrated activity that followed a power law (Takashima et al., 2006; Tallman et al., 2022b), a pattern that corresponds to normal forgetting (Wickelgren, 1974; Wixted and Carpenter, 2007).

First, four vectors of interest were created that coded for the target trials for each memory age condition (1 hour, 1 day, 1 week, 1 month). A fifth vector coded for foil trials. Second, beta coefficients for each memory age condition were obtained from multiple regression analysis for each participant. Third, we carried out a group-level linear mixed effect model (LME) analysis to examine voxels where the beta coefficients changed across time periods according to a power function (*y* = x^–0.33^) (Chen et al., 2013).

##### 2.4.5.2 Secondary analyses

The brain regions identified as changing as a function of memory age in the primary analysis could instead reflect changes in behavior or encoding/re-encoding, that also change with memory age. Therefore, we also carried out two secondary analyses to validate that our primary findings reflected memory retrieval.

**FIGURE 2:**
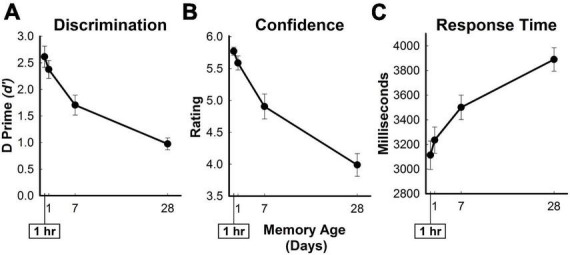
In-scanner behavioral measures of memory retrieval for three-word sentences. Average in-scanner recognition memory performance for all targets as a function of memory age. **(A)** Measure of discrimination (d’ [D Prime]) between old and new sentences. **(B)** Confidence ratings from 1 (definitely new) to 6 (definitely old) for targets. **(C)** Response time in milliseconds for targets. Error bars show SEM.

Amplitude-modulated analysis: The purpose of the amplitude-modulated analysis was to minimize the impact of concomitant changes in behavior on retrieval-related brain activity identified in the primary analysis. The in-scanner behavioral measures of memory resemble the predicted pattern of brain function and could interfere with the accurate detection of retrieval-related activity patterns (reported below, see Figure 2). As a result, the identified monotonic patterns of brain activity could be tracking changes in behavior rather than changes in memory age. Therefore, we conducted an additional multiple regression analysis which allowed for the examination of all trials while minimizing the trial-by-trial effects of behavior (as in Tallman et al., 2022b). The same multiple regression from the primary analysis was used with the addition of 8 new vectors, two new vectors for each of the four memory age condition which corresponded to trial-level memory confidence ratings and trial-level response times (*1dMarry*). The resulting beta coefficients associated with each memory age condition represented retrieval-related activity when the effects of the behavioral changes were minimized. The amplitude-modulated beta coefficients associated with each memory age condition were used in a second LME model identical to the primary analysis to obtain retrieval-related brain regions associated with memory age while minimizing the effects of behavior.

Re-encoding analysis: Another potential confound when detecting changes in retrieval-related activity associated with memory age is the presence of encoding-related activity in the same brain regions. Due to forgetting across the memory age conditions (reported below, see Figure 2), more re-encoding would be experienced for weakly remembered targets in the more remote conditions when compared to strongly remembered targets in the more recent conditions. Therefore, differential levels of re-encoding-related activity would appear as increases in retrieval-related brain activity associated with memory age.

We identified brain regions associated with successful encoding during the experiment by testing subsequent memory for in-scanner foils (post-test targets) during a surprise post-test (see Figure 1C). We created two vectors to define in-scanner foils as subsequently remembered or subsequently forgotten based on post-test responses. A multiple regression analysis was carried out with one vector coding for all target sentences, one vector coding for subsequently remembered in-scanner foils, and one vector coding for subsequently forgotten in-scanner foils. The subsequently forgotten beta coefficients for six subjects were excluded from analysis because they performed so well that they had 5 or fewer forgotten items according to the post-test. Thus, the re-encoding analysis included the subsequently remembered beta coefficients for all participants (*n* = 24) and the subsequently forgotten beta coefficients for a subset of subjects (*n* = 18). There were 89 ± 4.6 subsequently remembered in-scanner foil trials and 31 ± 4.6 subsequently forgotten in-scanner foil trials per participant, when excluding those 6 participants. Because LME can tolerate missing data, we carried out group-level analysis of encoding-related activity using *3dLME* to contrast subsequently remembered and subsequently forgotten foils (successful encoding: remembered >forgotten).

##### 2.4.5.3 Probability threshold and correcting for multiple comparisons

Significant clusters were identified using a voxel-wise probability value of *p* < 0.001 and a cluster-wise probability of *p* < 0.05 (Woo et al., 2014). This probability threshold reflects an optimized and robust approach to identifying clusters. These probabilities were entered into AFNI’s *3dClusterize* to determine the minimum cluster size needed to correct for multiple comparisons (16 voxels, 128 microliters). All cluster labels are reported in the tables using the gyrus and region information from the Brainnetome atlas (Fan et al., 2016).

#### 2.4.6 Whole-brain, voxel-wise analysis of functional connectivity

Generalized psychophysiological interaction (gPPI) analyses were carried out to examine functional connectivity changes associated with memory age. gPPI analyses are used to test the interaction between different psychological contexts and the relationship between a neural response in a particular seed region and the rest of the brain. (Friston et al., 1997; McLaren et al., 2012; Cisler et al., 2014). Thus, it is possible to assess whether variations in functional connectivity between a specific brain region (seed region) and the rest of the brain are associated with differences in levels of a psychological context. Alternatively, this interaction can be understood as the impact of different levels of a psychological context on the variations between the seed region and the rest of the brain.

##### 2.4.6.1 Creation of seed regions

We created gPPI models to examine the interaction between different levels of memory age across 1 hour to 1 month and the functional connectivity between the *a priori* ROIs (hippocampus and vmPFC) and the rest of the brain. The hippocampal analysis used the bilateral anatomical ROI seed region, described above. For the vmPFC gPPI analysis, an anatomical vmPFC seed region was selected from a previous study (Takashima et al., 2006) where an increase in activity was observed as memory age increased from 1 day to 3 months. To create the vmPFC seed region, an 8 mm sphere was dilated around their MNI coordinate (−2, 32, 10).

##### 2.4.6.2 Pre-processing for gPPI

Functional connectivity changes are detected at the neural level rather than detected within changes in the BOLD signal (Gitelman et al., 2003), therefore we prepared the BOLD signal from the primary analysis of memory age (described above) for gPPI modeling (McLaren et al., 2012; Cisler et al., 2014) using a series of AFNI commands (Chen, *Context-dependent correlation analysis or generalized PPI*).

1. For each functional run, estimates of baseline activity were obtained from the brain activation analysis GLM.
2. Baseline activity was subtracted from the concatenated and pre-processed functional runs.
3. For each seed region, a mean timecourse was created by averaging across all voxels in the ROI.
4. Neural interaction regressors were created by multiplying the seed region timecourse with the trial onset times for each memory age condition (1 hour, 1 day, 1 week, 1 month) and for foils, creating 5 vectors.
5. A GLM was carried out using the same model as in the primary activation analysis of memory age (see section 2.4.5.1), but with the addition of the neural interaction regressors and the seed region time course. The resulting four beta coefficients of the neural interaction regressors represent the magnitude of the effect of the seed region on the rest of the brain for each memory age, respectively.

For the primary analysis, the four beta coefficients from step 5 were examined using the same LME approach as was used in the brain activation analyses. This step tested whether hippocampal or vmPFC functional connectivity changed as a function of memory age. For the secondary analyses, steps 1–4 were carried out using the amplitude-modulated or successful encoding regression model as the starting point.

##### 2.4.6.3 Probability threshold and correcting for multiple comparisons

The same probability threshold and approach to correct for multiple comparisons for the brain activation analyses (primary and secondary) was used for the functional connectivity analyses. Because no significant clusters were detected at that threshold, we carried out an exploratory analysis at a more liberal probability threshold. We first examined the results at the exploratory probability threshold (voxel-wise *p* < 0.01, cluster-wise α = 0.05) from our previous paper (Tallman et al., 2022b) that had the same study design but used photos of indoor/outdoor scenes as memoranda. Because no clusters were detected with that voxel-wise threshold, we increased the threshold to *p* < 0.02, while maintaining a cluster-wise α = 0.05. These values were entered into 3dClusterize to identify the minimum cluster size (30 voxels, 240 microliters) needed for α = 0.05. Using this method revealed significant clusters for the primary analysis of hippocampal and vmPFC seeds. This threshold and minimum cluster size was applied to all secondary functional connectivity analyses.

## 3 Results

### 3.1 Behavioral findings

During the recognition memory test in the scanner, old-new discriminability (*d’*), response time, and confidence ratings decreased while response times increased across time periods (Figure 2). Participants obtained old-new discriminability scores of 2.6 ± 0.2, 2.4 ± 0.2, 1.7 ± 0.2, and 1.0 ± 0.1, average response times (ms) of 3112.8 ± 115.4, 3235 ± 106.8, 3501 ± 99.4, and 3889.5 ± 95.4, and confidence ratings of 5.8 ± 0.06, 5.6 ± 0.11, 4.9 ± 0.19, and 4.0 ± 0.18 for the hour, day, week, and month conditions, respectively. In-scanner behavioral measures significantly changed as a function of memory age [*d’*: *F*_(3,69)_ = 70.31, η^2^ = 0.754, *p* < 0.001; response time: *F*_(3,69)_ = 26.27, η^2^ = 0.533, *p* < 0.001; confidence ratings: *F*_(3,69)_ = 66.81, η^2^ = 0.744, *p* < 0.001]. Participants obtained 199.6 ± 5.9 hits (Hour: 57.7 ± 0.7; Day: 56.0 ± 1.3; Week: 48.7 ± 2.4; Month: 37.3 ± 2.4) and 40.4 ± 5.9 misses (Hour: 2.3 ± 0.7; Day: 4.0 ± 1.3; Week: 11.3 ± 2.4; Month: 22.8 ± 2.4). On the surprise post-scan recognition memory test, participants exhibited a *d’* of 2.0 ± 0.2.

### 3.2 Brain regions where activity changed as a function of memory age

**FIGURE 3:**
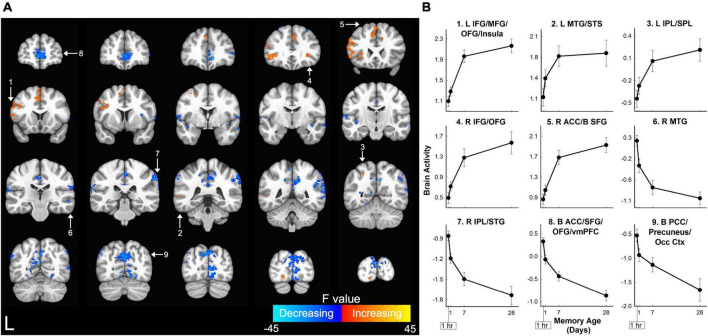
Memory age network in which retrieval-related brain activity for three-word sentences changed as a function of memory age. **(A)** Coronal sections displaying brain regions in which retrieval-related activity for sentences increased (warm colors) or decreased (cool colors) as memories aged from 1 hour to 1 month. Higher *F*-values (cyan or yellow) indicate activity that more closely followed a power function. Clusters corrected at voxel-wise *p* < 0.001, cluster-wise α = 0.05. White arrows and numbers highlight regions included in panel **(B)**. **(B)** Monotonic patterns of brain activity (beta coefficients) in selected regions from the frontal and parietal lobes shown in panel **(A)**. ACC, anterior cingulate cortex; B, bilateral; IFG, inferior frontal gyrus; IPL, inferior parietal lobule; L, left; MFG, middle frontal gyrus; MTG, middle temporal gyrus; Occ Ctx, occipital cortex; OFG, orbital frontal gyrus; PCC, posterior cingulate cortex; R, right; SFG, superior frontal gyrus; SPL, superior parietal lobule; STG, superior temporal gyrus; STS, superior temporal sulcus; vmPFC, ventral medial prefrontal cortex. Error bars show SEM.

The primary analysis detected a network of 30 brain regions within the prefrontal, temporal, parietal, and occipital cortices where activity was significantly associated with the age of the memory (Figure 3 and Table 1). Relatively monotonic increases or decreases in activity were observed in all brain regions. Regions increasing in activity as a function of memory age were identified in the bilateral prefrontal cortex (PFC), left middle temporal gyrus (MTG), and left superior parietal lobule (SPL). Regions decreasing in activity as a function of memory age were identified bilaterally in the prefrontal, temporal, parietal, and occipital cortices (Table 1).

**TABLE 1:** Brain regions where retrieval-related activity was associated with the age of the memory.

			MNI			
Brain region	Brainnetome atlas name	Vol (mm^3^)	X	Y	Z	B.A.
**Increasing activity with memory age**						
**Frontal**						
R Inf. Frontal/Orbital Frontal G.	R A12/47l, A44op	352	32	30	−1	12, 44, 47
L Inf./Mid. Frontal/Insular/Orbital Frontal/Precentral G.	L A12/47l, A44d, A44op, A44v, A45c, A45r, A6cvl, IFJ, IFS, dIa	6536	−50	22	16	6, 12, 44, 45, 47
R Cingulate G./B Sup. Frontal G.	R A32p, B A8m, B A9m	3608	−3	22	47	8, 9, 32
L Mid. Frontal G.	L A6vl	392	−29	−2	54	6
**Temporal**						
L Post. Sup. Temporal S./Mid. Temporal G.	L rpSTS	176	−54	−44	3	21
**Parietal**						
L Inf./Sup. Parietal Lob.	L A39rd, A7ip	176	−32	−60	51	7, 39
**Occipital**						
L Lat. Occipital Ctx.	L iOccG	352	−16	−92	−10	18
**Decreasing activity with memory age**						
**Frontal**						
B Ant. Cingulate/Orbital Frontal/Sup./Med. Frontal G.	B A10m, A14m, A32sg	7832	3	55	1	10, 14, 32
R Inf. Frontal G.	R A45r	304	51	43	4	45
R Mid./Sup. Frontal G.	R A8dl, A9/46d	216	28	34	50	8, 9, 46
L Sup. Frontal G.	R A8d	168	−25	29	57	8
**Temporal**						
R Sup. Temporal/Precentral G.	R A4tl, TE1.01.2	720	62	5	4	4, 6
R Insular/Precentral G.	R A4tl, dId	272	40	4	11	4, 13, 44
R Mid. Temporal G.	R aSTS	160	62	2	−18	21
L Sup. Temporal/Precentral G.	L A4tl, TE1.01.2	736	−62	−1	5	4, 6
L Sup. Temporal G.	L TE1.01.2	248	−45	−2	−11	22
R Insular G.	R vId/vIg	192	41	−5	−10	13
L Sup. Temporal/Postcentral G.	L A1/2/3tonIa, TE1.01.2	424	−61	−16	8	41
R Mid. Temporal G.	R aSTS	504	62	−17	−9	21
L Sup. Temporal G.	L TE1.01.2	272	−41	−18	−2	22
R Mid. Temporal G.	R A37dl	320	59	−63	8	37
**Parietal**						
L Inf. Parietal Lob./Sup. Temporal G.	L A22c, A40c, A40rv, A41/42	1584	−65	−34	26	22, 40–42
R Paracentral Lob.	R A1/2/3ll	168	14	−40	49	1–3
R Inf. Parietal Lob./Mid. Temporal G.	R A37dl, A39rv, A40c, A40rd, A40rv	7896	60	−43	32	37, 39, 40
L Paracentral Lob	L A5m	160	−7	−46	58	5
L Inf. Parietal Lob.	L A39rv	1504	−50	−72	36	39
L Inf. Parietal Lob.	L A39c	200	−39	−77	19	39
**Occipital**						
L MedioVentral Occipital Ctx.	L rLinG	304	−19	−64	−14	18
B Post. Cingulate G./Precuneus/Lat./MedioVentral Occipital Ctx./L Paracentral Lob.	L A1/2/3ll, B A23c, A23d, A31, A5m, A7m, OPC, cCunG, cLinG, dmPOS, msOccG, rCunG, rLinG, vmPOS	24904	2	−73	23	1–3, 5, 7, 23, 31
**Subcortical**						
L Post. Parietal White Matter	–	152	−33	−58	12	–

Activity in all clusters significantly changed across the four time periods according to a power function; voxel-wise threshold of *p* < 0.001, cluster-wise threshold of α = 0.05. For each lobe, clusters are listed from anterior to posterior based on the MNI coordinate of the center of mass. Names in the brain region column are the anatomical labels associated with the Brainnetome atlas code. Ant, anterior; BA, brodmann area; B, bilateral; Ctx, cortex; G, gyrus; Inf, inferior; L, left; Lat, lateral; Lob, lobule; Mid, middle; R, right; Post, posterior; S, sulcus; Sup, superior; Vol, volume.

### 3.3 Analysis of brain activity in hippocampal ROIs as a function of memory age

**FIGURE 4:**
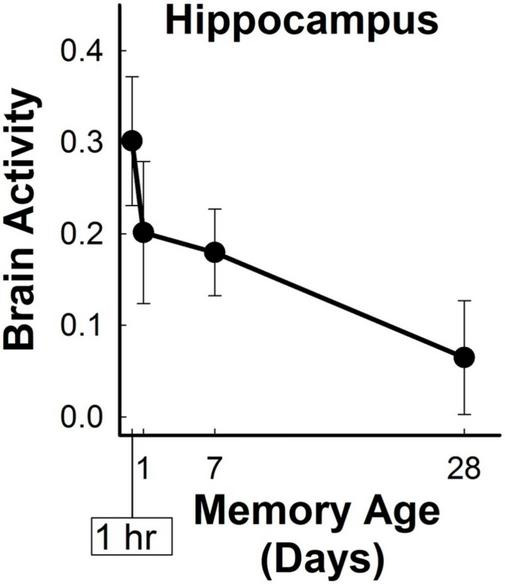
Hippocampal retrieval-related activity decreased for three-word sentences as a function of memory age. Bilateral hippocampal activity (beta coefficients) associated with the retrieval of target sentences significantly decreased from 1 hour to 1 month (*p* < 0.001).

Brain activity in the bilateral hippocampal anatomical ROI significantly changed with memory age according to a power function (LME Estimate: 0.075; SE: 0.02; df: 46.5; *t* = 3.97; *p* < 0.001) (Figure 4). Follow-up analyses to examine whether the effects of memory age were related to laterality or anterior/posterior portions of the hippocampus revealed similar findings. There were significant effects of memory age for the left, right, anterior, and posterior hippocampal ROIs (see Supplementary Table 1). Thus, activity in the entire hippocampus decreased as a function of memory age across 1 hour to 1 month. Note that a right posterior hippocampal cluster decreasing in activity as a function of memory age was observed in the whole-brain analysis described above when the cluster threshold was substantially more liberal (*p* < 0.02, α = 0.05; MNI center of mass: 18, −35, −15; volume = 696 mm^3^).

### 3.4 Secondary analyses to clarify the role of brain regions in the memory-age network

Two additional analyses were conducted to determine if the memory consolidation effects observed in the primary analysis were related to memory retrieval and not to additional confounding factors (see section 2.4.5.2). First, we conducted an amplitude-modulated analysis to determine if the changes in brain activity observed in the memory age were influenced by the concomitant behavioral changes associated with memory age (see Figure 2). All the brain regions associated with memory age in the primary analysis (see Table 1) were identified in the secondary analysis that minimized the effects of behavioral changes (Supplementary Table 2). There was substantial overlap in the voxels identified by the primary and secondary analyses of memory age (88%) (see Supplementary Figure 1) and no additional brain regions were identified. Generally, the memory age network and its patterns of activity remained unchanged and only varied based on the number of voxels identified in a particular brain region. The most notable differences were the identification of additional insular and lateral occipital voxels and the disappearance of middle temporal and PFC voxels after minimizing the effects of memory age. After minimizing the effects of behavior, activity in the bilateral hippocampal ROI still significantly decreased as a function of memory age (LME Estimate: 0.07; SE: 0.02; df: 46; *t* = 3.65 *p* < 0.001). Decreases in activity with memory age in the left, right, anterior, and posterior ROIs also remained significant. Therefore, the memory age network identified in the primary analysis did not appear to reflect concomitant changes in behavior associated with memory age.

Next, an analysis was conducted on the encoding-related activity associated with in-scanner foils to determine whether regions within the memory-age network overlapped with those involved in successful memory encoding. Two clusters (left caudate and right MFG/IFG) were identified where activity reflected successful encoding (Supplementary Table 3). Importantly, there was no voxel overlap between the memory-age network and this encoding network. Thus, the memory age network identified in the primary analysis reflected changes in retrieval associated with memory consolidation and not activity associated with encoding.

### 3.5 Brain regions where functional connectivity changed as a function of memory age

**FIGURE 5:**
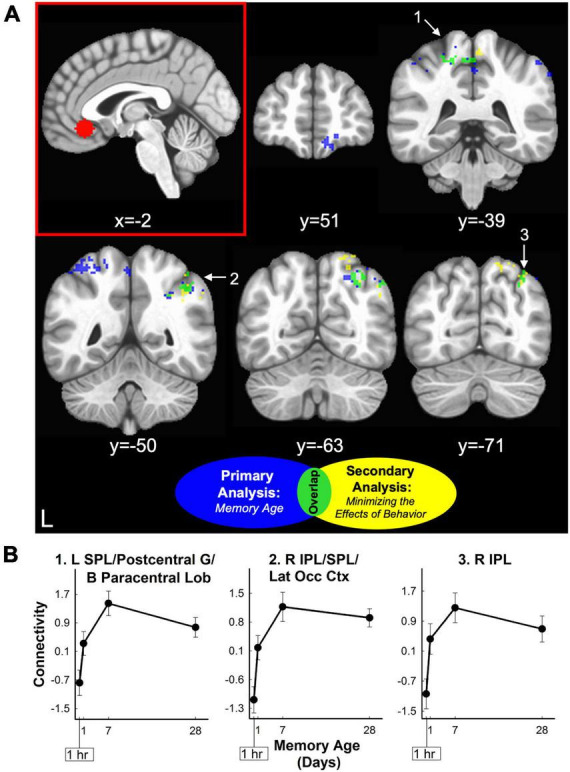
vmPFC retrieval-related functional connectivity increased for three-word sentences as a function of memory age. **(A)** Sagittal section displaying the vmPFC seed region used for functional connectivity analysis (red panel). Coronal sections displaying clusters where retrieval-related functional connectivity associated with the vmPFC seed region increased as a function of memory age from 1 hour to 1 month. Clusters were corrected at voxel-wise *p* < 0.02, cluster-wise α = 0.05. Clusters are colored to show results before (blue) and after (yellow) minimizing the effects of behavior (amplitude modulation). Three clusters (lime green) in the posterior parietal cortex were common to both analyses (see also Table 2). White arrows and numbers highlight regions included in panel **(B)**. **(B)** Monotonic patterns of functional connectivity (primary analysis of memory age beta coefficients) between the vmPFC and the three clusters in the posterior parietal cortex common to both analyses (lime green). These clusters continued to show a relatively monotonic increasing pattern of connectivity even when minimizing the effects of behavior (amplitude modulation; see also Table 2). B, bilateral; IPL, inferior parietal lobule; L, left; Lat Occ Ctx, lateral occipital cortex; Lob, lobule; R, right; SPL, superior parietal lobule.

**FIGURE 6:**
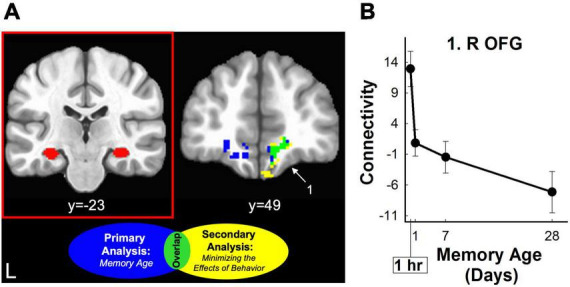
Hippocampal retrieval-related functional connectivity decreased for three-word sentences as a function of memory age. **(A)** Left Coronal section displaying an example seed region from one subject used for functional connectivity analysis (red panel). Right Coronal section displaying clusters where retrieval-related functional connectivity associated with the hippocampal seed region decreased as a function of memory age from 1 hour to 1 month. Clusters were corrected at voxel-wise *p* < 0.02, cluster-wise α = 0.05. Clusters are shown before (blue) and after (yellow) minimizing the effects of behavior (amplitude modulation). Only one cluster (lime green) in the OFG (part of vmPFC) was common to both analyses (see also Table 2) White arrow and number highlight region in panel **(B)**. **(B)** Monotonic pattern of functional connectivity (primary analysis of memory age beta coefficients) between the hippocampus and the right OFG common to both analyses (lime green). This cluster continued to show a monotonic decreasing pattern of connectivity even when minimizing the effects of behavior (amplitude modulation; see also Table 2). L, left; R, right; OFG, orbital frontal gyrus.

Analysis of vmPFC functional connectivity and hippocampal connectivity did not reveal any significant clusters where connectivity significantly changed with memory age (voxel-wise *p* < 0.001, cluster-wise α = 0.05). Exploratory analysis at a lower probability threshold (voxel-wise *p* < 0.02, cluster-wise α = 0.05) did identify brain regions that exhibited changes in functional connectivity with either the hippocampus or vmPFC. For the vmPFC, connectivity increased with right vmPFC, right orbital frontal cortex (OFC) (middle frontal and orbital gyri), and with the posterior parietal cortex (PPC) bilaterally (4 clusters covering superior and inferior parietal lobules, precuneus, and sensorimotor areas). After minimizing the effects of behavior (amplitude-modulation analysis), vmPFC

connectivity with the right posterior parietal cortex remained significant at the exploratory threshold (Table 2 and Figure 5). For the hippocampus, functional connectivity decreased with the left and right vmPFC (OFG, ACC) and increased with left cerebellum. After minimizing the effects of behavior (amplitude-modulation analysis), connectivity between the hippocampus and right vmPFC and the cerebellum remained significant at the exploratory threshold (Table 2 and Figure 6).

**TABLE 2:** Brain regions where retrieval-related functional connectivity was associated with the age of the memory.

			MNI				
Brain region	Brainnetome atlas name	Vol (mm^3^)	X	Y	Z	B.A.	M.P.
**vmPFC: non-AM connectivity**							
**Frontal**							
R Mid. Frontal/Orbital Frontal G.	R A10l, A11l, A11m	1464	20	62	−17	10, 11	↑
R Pre/Postcentral G.	R A1/2/3ulhf, A2, A4hf, A6cvl	1176	55	−8	45	1–4, 6	↑
**Parietal**						
L Inf./Sup. Parietal/Paracentral Lob./Pre/Postcentral G./B Precuneus	L A1/2/3ll, A1/2/3tru, A2, A40rd, A4ul, A5l, A7ip, A7pc B A5m	4264	−23	−43	59	1–5, 7, 40	↑
R Inf. Parietal Lob.	R A39rd, A39rv, A40c, A40rd	2104	50	−50	49	39, 40	↑
R Inf./Sup. Parietal Lob.	R A39rd, A7c, A7ip	1768	31	−62	49	7, 39	↑
**vmPFC: AM connectivity**							
**Parietal**							
B Paracentral Lob./L Postcentral G./Sup. Parietal Lob.	B A1/2/3ll, L A1/2/3tru, A7pc	1088	−14	−38	58	1–3, 7	↑
R Inf. Parietal Lob.	R A39rd, A39rv, A40c, A40rd	1608	48	−52	45	39, 40	↑
R Inf./Sup Parietal Lob./Lat. Occipital Ctx.	R A39rd, A7c, A7ip, A7r, lsOccG	1784	29	−64	53	7, 49	↑
**Hippocampus: non-AM connectivity**							
**Frontal**							
L Cingulate/Orbital Frontal G.	L A11l, A11m, A14m, A32sg	1136	−14	50	−11	11, 14, 32	↓
R Orbital Frontal G.	R A11m, A14m	1064	10	42	−11	11, 14	↓
**Subcortical**							
L Cerebellum	–	1336	−20	−78	−35	–	↑
**Hippocampus: AM connectivity**							
**Frontal**							
R Orbital Frontal G.	R A11m, A14m	1600	11	43	−15	11, 14	↓
**Subcortical**							
L Cerebellum	–	1760	−22	−77	−33	–	↑

Functional connectivity significantly changed across the four time periods according to a power function (voxel-wise threshold of *p* < 0.02, cluster-wise threshold of α = 0.05). AM (amplitude-modulated analysis) indicates that connectivity changed across time periods when the effect of concomitant changes in behavior were minimized. For each seed (ventromedial prefrontal cortex [vmPFC] or hippocampus), functional connectivity changed in a relatively monotonic pattern (M.P.) across time periods [M.P., increasing (↑) or decreasing (↓)]. Names in the brain region column are the anatomical labels associated with the Brainnetome atlas code. For each lobe, clusters are listed from anterior to posterior based on the MNI coordinate of the center of mass. AM, amplitude-modulated; Ant, anterior; BA, brodmann area; B, bilateral; Ctx, cortex; G, gyrus; Inf, inferior; L, left; Lat, lateral; Lob, lobule; Mid, middle; R, right; Post, posterior; S, sulcus; Sup, superior; Vol., volume.

To determine if there was overlap between the retrieval-related connectivity described above and encoding-related connectivity (subsequently remembered in-scanner foils vs. subsequently forgotten in-scanner foils), we assessed the overlap between these two analyses using the same exploratory cluster probability threshold. When using the hippocampus as a seed region, no significant clusters were identified where connectivity reflected successful encoding (see Supplementary Table 4). When using the vmPFC as a seed region, four significant clusters spanning the bilateral temporal cortex (left hippocampus, bilateral fusiform, superior, and inferior temporal gyrus), bilateral temporoparietal junction (posterior STS and IPL), and right lateral occipital cortex were identified where connectivity was related to successful encoding (see Supplementary Table 5). There was no overlap between these clusters and the clusters identified by the memory age vmPFC-PPC connectivity results. Therefore, the functional connectivity results related to memory age and did not reflect encoding or changes in behavior.

## 4 Discussion

We examined changes in retrieval-related brain activity and functional connectivity associated with the long-term memory consolidation of three-word sentences from 1 hour to 1 month. In-scanner behavioral measures of discriminability, confidence ratings, and response time significantly changed as a function of memory age. Primary analyses identified a widespread network of neocortical regions that demonstrated relatively monotonic increases or decreases in retrieval-related activity associated with memory age (Figure 3 and Table 1). Hippocampal brain activity within an anatomical ROI significantly decreased with memory age (Figure 4), regardless of whether we examined left, right, anterior, or posterior portions. Functional connectivity of the hippocampus or vmPFC did not significantly change as a function of memory age, although we observed patterns of changes consistent with memory consolidation at a less stringent voxel-wise, but a similar cluster-wise threshold (Figures 5, 6 and Table 2). Secondary analyses that examined if the primary retrieval-related brain activity and functional connectivity changes reflected confounding factors (changes in behavior with memory age or re-encoding of targets) revealed that our primary findings remained when these confounding factors were taken into account (Supplementary Figure 1 and Supplementary Tables 2–5).

### 4.1 Changes in retrieval-related brain activity associated with long-term memory consolidation

#### 4.1.1 Patterns of cortical brain activity as a function of memory age

Systems consolidation theory posits that as time passes after learning, long-term memories are initially dependent on the hippocampus and are slowly stabilized in the cortex until they can eventually be retrieved independently of the MTL (Marr, 1971; McClelland et al., 1992, 1995; Alvarez et al., 1994). As memories become established in the cortex over time, this process can be reflected as increases in fMRI brain activity as memories age. We identified a memory age network which exhibited increased brain activity associated with memory age within the prefrontal, temporal, posterior parietal, and lateral occipital cortices. Several regions of this network overlapped with regions previously identified as showing increasing activity with memory age for verbal memory, including the left PFC (left MFG, SFG, right ACC), left SPL, right STS, and bilateral IPL (Bosshardt et al., 2005a,b). Voxel-wise overlap across studies examining changes in activity over time is difficult to ascertain due to the variation in experimental designs and memoranda studied. Nevertheless, at the level of brain regions, increases are consistently observed over relatively short time periods, ranging from 1 hour to several months in the prefrontal and parietal cortex (Takashima et al., 2006, 2009; Gais et al., 2007; Suchan et al., 2008; Davis et al., 2009; Sterpenich et al., 2009; Yamashita et al., 2009). More specifically, a previous study of memory for indoor/outdoor scenes used a similar design as the current study and identified the same regions. It showed increasing activity in bilateral MFG, SPL, precuneus, sensorimotor regions, left insula, and associative visual cortex (Tallman et al., 2022b).

Contrary to the predictions of systems consolidation theory, we also observed widespread decreases in brain activity as a function of memory age. While it remains unclear exactly why certain cortical brain regions exhibit reduced in retrieval-related activity over time, this finding was observed in several studies of memory consolidation over shorter time periods, specifically within the prefrontal and parietal cortex (Bosshardt et al., 2005b; Harand et al., 2012; Takashima et al., 2017). Consistent with our earlier study of memory for indoor/outdoor scenes across the same intervals (Tallman et al., 2022b), we observed decreases in the bilaterally in mPFC, MFG, PPC, PCC, IPL, precuneus, and left SFG.

#### 4.1.2 Patterns of hippocampal brain activity as a function of memory age

The consensus for patterns of hippocampal brain activity associated with memory age over short time periods (e.g., hours to several months) is as sparse as the experimental designs, types of memoranda tested, and time periods examined are variable. This variability poses difficulties in determining whether time-dependent changes support a specific memory consolidation theory and whether it is possible to detect time-dependent changes in memory retrieval across short time periods. Several studies found decreases in hippocampal activity associated with memory age, supporting SCT across intervals greater than ∼60 days (Takashima et al., 2006; Sterpenich et al., 2009; Milton et al., 2011; Furman et al., 2012; Harand et al., 2012), between ∼30 and ∼40 days (Smith et al., 2010; Dandolo and Schwabe, 2018; Du et al., 2019), and less than ∼30 days (Bosshardt et al., 2005b; Takashima et al., 2009; Ritchey et al., 2015; Sekeres et al., 2018). Like these other studies, the current study (28 day interval), also identified decreases in retrieval-related hippocampal activity, thus supporting the predictions of SCT. Note that decreases in activity may also be taken as support for TT and CBT because these decreases are thought to reflect forgetting of detail and/or contextual information, but many of the above studies did not measure these characteristics of memory.

Multiple-trace/transformation theory predicts that the repeated retrieval of an episodic memory increases the number of memory traces within the hippocampus, thus increases in retrieval-related hippocampal activity are interpreted as support for this process. Other studies have observed increases in retrieval-related activity associated with memory age ranging across intervals of ∼200 days (Vanasse et al., 2022), ∼30 days (Bosshardt et al., 2005a; Smith et al., 2010) and less than 2 days (Bosshardt et al., 2005b; Gais et al., 2007). Even more studies found no change in retrieval-related hippocampal activity associated with memory age at intervals up to ∼45 days (Stark and Squire, 2000; Janzen et al., 2008; Suchan et al., 2008; Davis et al., 2009; Vilberg and Davachi, 2013; Tompary and Davachi, 2017; Du et al., 2019; Tallman et al., 2022b). It is possible no change in retrieval-related hippocampal activity reflects the persistent hippocampal engagement in memory retrieval across all time periods, as predicted by MTT/TT. CBT also posits that no change in hippocampal activity could reflect sustained hippocampal engagement when memory retrieval involves contextual information. Alternatively, null results could be due a lack of statistical power to detect time-dependent changes in hippocampal activity. However, many of these studies, including the current study, were not designed to adjudicate between the different memory consolidation theories. Relevant characteristics of the retrieved memories, such as the number of details, vividness, and specificity, should be examined in future studies to directly test these theories.

When considering only studies of verbal material, the picture is not much clearer. Like the findings reported here, three studies also reported decreases in hippocampal activity associated with memory age, supporting SCT (left hippocampus: Bosshardt et al., 2005b; Ritchey et al., 2015; associative: Du et al., 2019). Conversely, three studies found increases in hippocampal activity associated with memory age, aligning with MTT/TT predictions (Bosshardt et al., 2005a; right hippocampus: Bosshardt et al., 2005b; Gais et al., 2007). Two studies did not detect significant changes in hippocampal activity associated with memory age (Davis et al., 2009; item memory: Du et al., 2019). The current study provides evidence that hippocampal activity patterns consistent with SCT predictions can be observed for verbal material over short time periods, although the consensus of hippocampal activation patterns across similar studies remains mixed.

### 4.2 Changes in retrieval-related functional connectivity associated with long-term memory consolidation

#### 4.2.1 Patterns of cortico-cortical functional connectivity as a function of memory age

Systems consolidation theory predicts changing *connections* between brain regions, suggesting functional connectivity measures may be more apt for detecting long-term memory consolidation effects relative to brain activity measures. Specifically, SCT proposes that both existing and new cortico-cortical functional connections strengthen over time. The vmPFC is thought to be the “integration” hub, taking over memory retrieval functions from the hippocampus via communication with the rest of the cortex. This account suggests that vmPFC-cortical connectivity should increase with memory age. Exploratory analysis of vmPFC functional connectivity was consistent with this prediction, showing increased connectivity with vmPFC/OFC and PPC with memory age. The finding that vmPFC-PPC connectivity increases with memory age, replicates the finding for memory of indoor/outdoor scenes across the same time period (Tallman et al., 2022b). These interactions between the vmPFC and PPC occur rapidly and are thought to establish memory traces in the PPC (Brodt et al., 2016, 2018).

#### 4.2.2 Patterns of hippocampal-cortical functional connectivity as a function of memory age

According to standard SCT, bidirectional connections between the MTL and neocortex change as a function of memory age to reorganize memory traces, enabling the retrieval of long-term memories without the MTL (Squire and Alvarez, 1995). SCT predicts the connections between the MTL and neocortex weaken over time, possibly reflected as decreases in functional connectivity as a function of memory age. We identified such decreases in functional connectivity between the hippocampus and the vmPFC and OFC over a 1-month period, albeit at an exploratory voxel-wise threshold. This finding is consistent with connectivity findings from previous studies of long-term memory consolidation across this time period (Takashima et al., 2006; van Kesteren et al., 2010) and longer time periods spanning years to decades (Söderlund et al., 2012). Notably, in our prior study, we did not detect changes in hippocampal-vmPFC connectivity, but we did observe decreased functional connectivity between the hippocampus and specific brain regions, including the right medial frontal gyrus (MFG) and lateral temporal cortex (Tallman et al., 2022b).

The vmPFC acts as mediator between hippocampus and cortex to integrate memories and stabilize them in the neocortex (Bontempi et al., 1999; Frankland and Bontempi, 2005; Nieuwenhuis and Takashima, 2011). Consequently, as the role of the vmPFC and its cortical connections strengthen over time, the role of the hippocampus and its cortical connections diminishes over time. Taken together, our functional connectivity findings demonstrate a simultaneous reorganization of the connections between the hippocampus, vmPFC, and cortex, supporting the predictions of systems consolidation theory. Because our findings were identified using an exploratory voxel-wise threshold, future studies are needed to identify further evidence for memory reorganization using measures of functional connectivity.

## 5 Summary

In summary, we identified changes in retrieval-related brain activity and functional connectivity for three-word sentences as a function of memory age from 1 hour to 1 month. Time-dependent increases and decreases in activation were observed in a widespread cortical memory age network. Hippocampal activity within an anatomical ROI significantly decreased with memory age. Cortico-cortical functional connectivity increased while hippocampal-cortical functional connectivity decreased as a function of memory age, albeit at a less stringent voxel-wise threshold. These concurrent findings (changes in activity and connectivity) were predicted by SCT and are in line with the idea that long-term memory consolidation effects can be detected over a short time period. In order to adjudicate whether such findings support SCT or more recent theories (e.g., MTT/TT, CBT), future studies are needed that assess how particular characteristics of retrieved memories (e.g., number of details, vividness, specificity) may influence changes in brain activity and connectivity associated with memory age.

## Data availability statement

The raw data supporting the conclusions of this article will be made available by the authors, without undue reservation.

## Ethics statement

The studies involving humans were approved by the Research and Development Committee, Veterans Affairs San Diego Healthcare System. The studies were conducted in accordance with the local legislation and institutional requirements. The participants provided their written informed consent to participate in this study.

## Author contributions

CT: Data curation, Formal Analysis, Investigation, Methodology, Validation, Visualization, Writing – original draft, Writing – review and editing. ZL: Formal Analysis, Software, Writing – review and editing. CS: Conceptualization, Data curation, Formal Analysis, Funding acquisition, Investigation, Methodology, Project administration, Resources, Supervision, Validation, Visualization, Writing – original draft, Writing – review and editing.
