# Supplementary material for: Human brain activity and functional connectivity associated with verbal long-term memory consolidation across 1 month
Source: Front Hum Neurosci. 2024 Feb 21;18:1342552. doi: 10.3389/fnhum.2024.1342552 (PMC10915245; doi:10.3389/fnhum.2024.1342552)
Supplement: Supplementary file 1 [file Data_Sheet_1.docx]

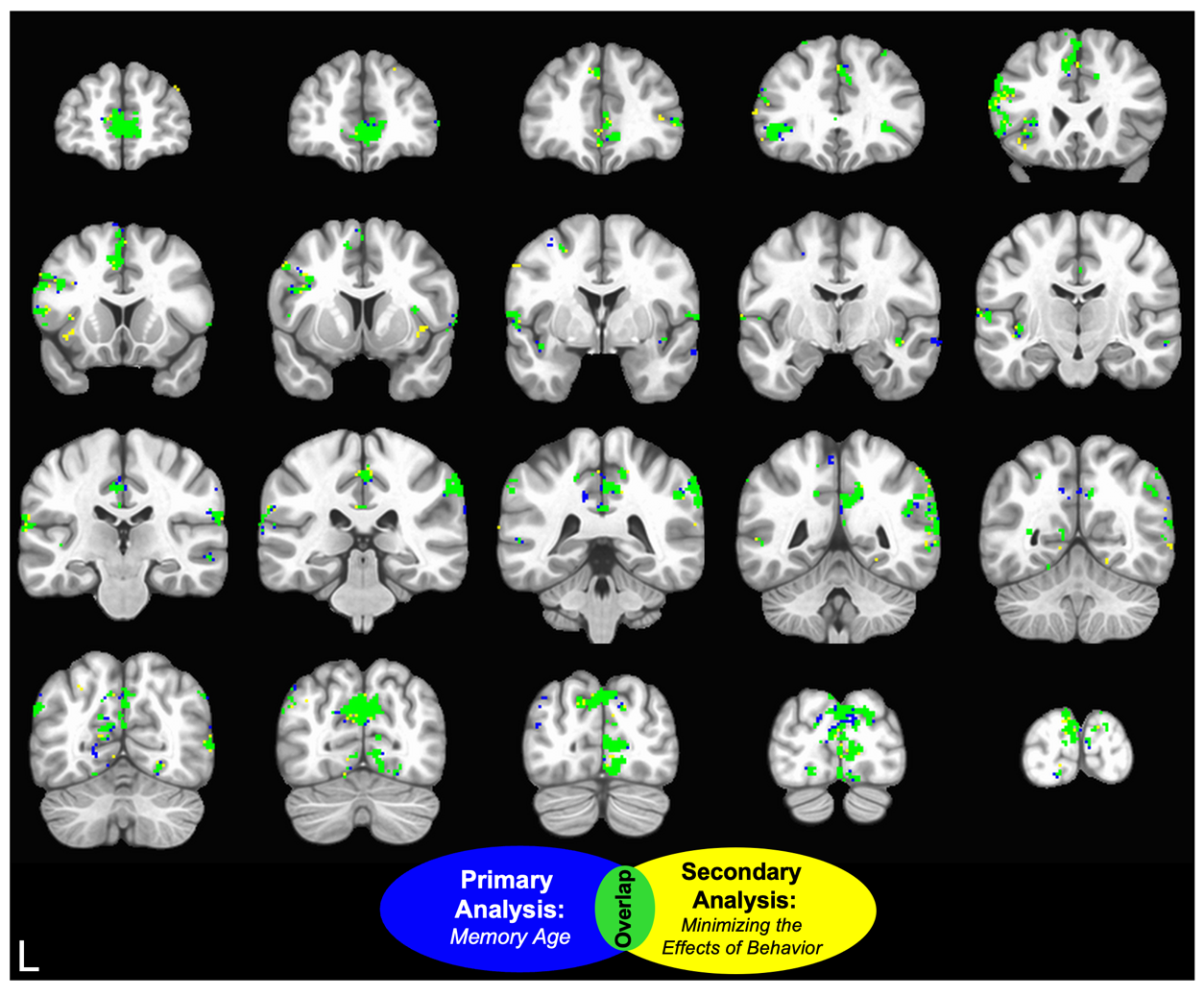


**Supplementary Figure 1.** Coronal sections displaying overlap between brain activation from the memory age network and the memory age network after minimizing the effects of behavior. Retrieval-related activation from the primary analysis of memory age (blue) is the same network seen in Figure 3 (see Table 1 for a list of brain regions.). Retrieval-related activation from the secondary analysis, which used amplitude-modulation to minimize the effects of behavior (yellow), identified all the brain regions from the primary analysis. There was 88% direct voxel overlap between the primary and secondary analysis of the memory age brain activation network.

| Supplementary Table 1. Retrieval-related brain activity changes in hippocampal regions of interest | | | | | |
| --- | --- | --- | --- | --- | --- |
|  | Linear Mixed Effects Analysis | | | | |
| ROI | Estimate | SE | df | t value | p value |
|  | *Hippocampal non-AM brain activity* | | | | |
| Bilateral | 0.075 | 0.019 | 47 | 3.97 | 0.0003 |
| Left | 0.075 | 0.027 | 23 | 2.79 | 0.01 |
| Right | 0.069 | 0.020 | 52 | 3.42 | 0.001 |
| Anterior | 0.065 | 0.027 | 34 | 2.39 | 0.02 |
| Posterior | 0.084 | 0.020 | 65 | 4.31 | 0.00006 |
|  | *Hippocampal AM brain activity* | | | | |
| Bilateral | 0.070 | 0.019 | 46 | 3.65 | 0.0007 |
| Left | 0.071 | 0.028 | 23 | 2.52 | 0.02 |
| Right | 0.064 | 0.020 | 55 | 3.25 | 0.002 |
| Anterior | 0.061 | 0.028 | 35 | 2.19 | 0.04 |
| Posterior | 0.079 | 0.020 | 53 | 3.83 | 0.0003 |
| *Note*. Significant brain activity changes in anatomical hippocampal regions of interest (ROI). Bilateral hippocampal ROIs were created using FSL FIRST to segment each participant’s T1-weighted image co-registered to MNI space. The anterior and posterior hippocampus was defined by the MRI slice where the uncus was no longer visible and demarcated the beginning of the posterior hippocampal ROI. A linear mixed effect model tested whether brain activity changed according to a power function. | | | | | |

| Supplementary Table 2. Brain regions where retrieval-related activity was associated with the age of the memory after minimizing the effects of behavioral changes | | | | | | | | |  |  |
| --- | --- | --- | --- | --- | --- | --- | --- | --- | --- | --- |
|  |  | Vol (mm^3^) | MNI | | |  | |  | | |
| Brain Region | Brainnetome Atlas Name |  | X | Y | Z | | B.A. | M.P. | |  |
| *Increasing activity with memory age* |  |  |  |  |  | |  |  | |  |
| Frontal |  |  |  |  |  | |  |  | |  |
| R Inf. Frontal/Frontal Orbital G. | R A12/47l, A44op | 368 | 32 | 30 | -2 | | 12, 44, 47 | ↑ | |  |
| L Inf./Mid. Frontal/Insular/Frontal Orbital G. | L A12/47l, A44d, A44op, A44v, A45c, A45r, IFJ, IFS, dIa | 6664 | -51 | 23 | 14 | | 12, 44-45, 47 | ↑ | |  |
| R Cingulate/B Sup. Frontal G. | R A32p, B A8m, A9m | 3664 | -3 | 22 | 47 | | 8, 32 | ↑ | |  |
| L Mid. Frontal/Precentral G. | L A6cvl, IFJ | 488 | -40 | 8 | 30 | | 6 | ↑ | |  |
| L Mid Frontal/Precentral G. | L A6cvl | 184 | -55 | 5 | 41 | | 6 | ↑ | |  |
| L Mid. Frontal G. | L A6vl | 272 | -26 | -3 | 52 | | 6 | ↑ | |  |
| Temporal |  |  |  |  |  | |  |  | |  |
| L Post. Sup. Temporal S./Mid. Temporal G. | L rpSTS | 160 | -54 | -45 | 3 | | 21 | ↑ | |  |
| Parietal |  |  |  |  |  | |  |  | |  |
| L Inf./Sup. Parietal Lob. | L A39rd, A7ip | 256 | -31 | -61 | 49 | | 7, 39 | ↑ | |  |
| Occipital |  |  |  |  |  | |  |  | |  |
| L Lat. Occipital Ctx. | L iOccG | 312 | -17 | -91 | -10 | | 18 | ↑ | |  |
| *Decreasing activity with memory age* |  |  |  |  |  | |  |  | |  |
| Frontal |  |  |  |  |  | |  |  | |  |
| B Ant. Cingulate/Frontal Orbital/Sup. Frontal G. | B A10m, A14m, A32sg | 7728 | 3 | 55 | 1 | | 10, 14, 32 | ↓ | |  |
| R Inf. Frontal G | R A45r, IFS | 392 | 49 | 42 | 5 | | 45 | ↓ | |  |
| L Sup. Frontal G. | L A8dl | 144 | -25 | 28 | 57 | | 8 | ↓ | |  |
| R Mid. Frontal G. | R A9/46d | 216 | 28 | 33 | 50 | | 9, 46 | ↓ | |  |
| R Sup. Frontal G. | R A9/46d | 176 | 25 | 51 | 40 | | 9 | ↓ | |  |
| R Sup. Temporal/Precentral G. | R A4tl, TE1.01.2 | 608 | 62 | 5 | 5 | | 4 | ↓ | |  |
| L Insular/Sup. Temporal G. | L A38l, Vl/vlg, TE1.0 | 232 | -44 | -3 | -11 | | 21 | ↓ | |  |
| L Sup. Temporal/Pre/Postcentral G. | L A1/2/3tonIa, A4tl, TE1.01.2 | 1072 | -62 | -6 | 6 | | 1-4 | ↓ | |  |
| Temporal |  |  |  |  |  | |  |  | |  |
| R Insular G. | R dId | 160 | 39 | 9 | -3 | | 13 | ↓ | |  |
| R Insular/Precentral G. | R A4tl, dId | 232 | 39 | 5 | 11 | | 4,21 | ↓ | |  |
| R Insular G. | R vId/vIg | 232 | 41 | -5 | -10 | | 13 | ↓ | |  |
| L Sup. Temporal G. | L TE1.01.2 | 280 | -41 | -18 | -2 | | 41 | ↓ | |  |
| R Mid. Temporal G. | R aSTS | 232 | 60 | -21 | -10 | | 21 | ↓ | |  |
| R Mid. Temporal G. | R A37dl | 376 | 59 | -63 | 8 | | 37 | ↓ | |  |
| Parietal |  |  |  |  |  | |  |  | |  |
| B Post. Cingulate G. | B A23c | 504 | -2 | -22 | 40 | | 23 | ↓ | |  |
| R Inf. Parietal Lob. | R A40rv | 672 | 66 | -23 | 20 | | 40 | ↓ | |  |
| L Inf. Parietal Lob./Sup. Temporal G. | L A22c, A40c, A40rv, A41/42 | 1480 | -65 | -34 | 26 | | 22, 40-42 | ↓ | |  |
| R Paracentral Lob. | R A1/2/3ll | 208 | 14 | -39 | 51 | | 1-3 | ↓ | |  |
| B Post. Cingulate G/Paracentral Lob./Precuneus | B A1/2/3ll, A23c, A31, A23d, L A5m | 3600 | 3 | -44 | 37 | | 1-3, 5, 23, 31 | ↓ | |  |
| R Inf. Parietal Lob./Mid. Temporal G | R A37dl, A39rv, A40c, A40rd, A40rv | 7072 | 60 | -45 | 33 | | 37, 39, 40 | ↓ | |  |
| L Precuneus | L A31 | 224 | -13 | -51 | 3 | | 31 | ↓ | |  |
| L Post. Cingulate G. | L A23v | 144 | -15 | -54 | 3 | | 23 | ↓ | |  |
| L Inf. Parietal Lob. | L A39rv | 1304 | -53 | -71 | 36 | | 39 | ↓ | |  |
| L Precuneus/Inf. Parietal Lob. | L A39rv | 144 | -40 | -85 | 39 | | 39 | ↓ | |  |
| Occipital |  |  |  |  |  | |  |  | |  |
| R MedioVentral Occipital Ctx. | R rLinG | 160 | 21 | -53 | -9 | | 18 | ↓ | |  |
| L MedioVentral Occipital Ctx. | L rLinG | 280 | -18 | -64 | -14 | | 18 | ↓ | |  |
| R MedioVentral Occipital Ctx. | R rLinG | 512 | 24 | -69 | -9 | | 18 | ↓ | |  |
| B Precuneus/Lat./MedioVentral Occipital Ctx. | B A31, A7m, OPC, cCunG, cLinG, BmPOS, msOccG, rCunG, rLinG, vmPOS | 18856 | 2 | -81 | 20 | | 7, 18, 31 | ↓ | |  |
| Subcortical |  |  |  |  |  | |  |  | |  |
| L Post. Parietal White Matter | -- | 160 | -32 | -58 | 12 | | -- | ↓ | |  |
| *Note*. Activity in all clusters significantly changed across the four time periods according to a power function; voxel-wise threshold of p < 0.001, cluster-wise threshold of p < 0.05. For each monotonic pattern (M.P.) of activity across time periods [increasing (↑) or decreasing (↓)], clusters are listed from anterior to posterior based on the MNI coordinate of the center of mass. Names in the brain region column are the anatomical labels associated with the Brainnetome atlas name. Ant. Anterior; BA., Brodmann area; B, Bilateral; Ctx., Cortex; G., Gyrus; Inf., Inferior; L, Left; Lat., Lateral; Lob., Lobule; Mid., Middle; R, Right; Post., Posterior; S. Sulcus; Sup., Superior; Vol., Volume. | | | | | | | | | | |

| Supplementary Table 3. Brain regions where encoding-related activity distinguished subsequently remembered from subsequently forgotten foils | | | | | | | |
| --- | --- | --- | --- | --- | --- | --- | --- |
|  |  | Vol  (mm^3^) | MNI | | |  |  |
| Brain Region | Brainnetome Atlas Name |  | X | Y | Z | B.A. | M.P. |
| *Successful Encoding (Remembered > Forgotten)* | | | | | | | |
| Frontal |  |  |  |  |  |  |  |
| R Mid./Inf. Frontal G. | R A44d, A8vl, A9/46v, IFS | 176 | 45 | 32 | 21 | 8,9,46 | ↑ |
| Subcortical |  |  |  |  |  |  |  |
| L Caudate | L dCa | 128 | -13 | 6 | 11 | - | ↑ |
| *Unsuccessful Encoding (Forgotten > Remembered)* | | | | | | | |
| Temporal |  |  |  |  |  |  |  |
| R. Inf. Temporal G. | R A20r | 144 | 46 | -4 | -46 | 20 | ↓ |
| Parietal |  |  |  |  |  |  |  |
| L Inf. Parietal Lob. | L A40c | 136 | 65 | -38 | 42 | 40 | ↓ |
| L Inf. Parietal Lob. | L A40c, A40v | 136 | -59 | -42 | 36 | 40 | ↓ |
| L Inf. Parietal Lob. | LA39rd, A39rv, A40c | 192 | -47 | -53 | 38 | 39,40 | ↓ |
| L Inf. Parietal Lob. | L A40c | 136 | -56 | -56 | 42 | 40 | ↓ |
| Occipital |  |  |  |  |  |  |  |
| L Lat./MedioVentral Occipital Ctx./Precuneus | L dmPOS, lsOccG, vmPOS | 136 | -18 | -72 | 31 | 7 | ↓ |
| L Fusiform G/ Lat./MedioVentral Occipital Ctx. | L A37mv, cLinG, iOccG | 184 | -20 | -83 | -15 | 37 | ↓ |
| Subcortical |  |  |  |  |  |  |  |
| L Cerebellum | - | 152 | -31 | -39 | -40 | - | ↓ |
| R Cerebellum | - | 176 | 22 | -69 | -35 | - | ↓ |
| L Cerebellum | - | 384 | -22 | -73 | -33 | - | ↓ |
| Note. Significant clusters reflected successful encoding (remembered > forgotten) or unsuccessful encoding (forgotten > remembered) of in-scanner foils (voxel-wise threshold of p < 0.001, cluster-wise threshold of p < 0.05). Names in the brain region column are the anatomical labels associated with the Brainnetome atlas code. BA., Brodmann area; B, Bilateral; Ctx., Cortex; G., Gyrus; Inf., Inferior; L, Left; Lat., Lateral; Lob., Lobule; Mid., Middle; M.P., Monotonic pattern increasing (↑) or decreasing (↓)]; R, Right; Vol., Volume. | | | | | | | |

| Supplementary Table 4. Brain regions where encoding-related hippocampal connectivity distinguished subsequently remembered from subsequently forgotten foils | | | | | | | | |  |
| --- | --- | --- | --- | --- | --- | --- | --- | --- | --- |
|  |  | Vol  (mm^3^) | MNI | | |  | |  | |
| Brain Region | Brainnetome Atlas Name |  | X | Y | Z | | B.A. | M.P. | |
| *Successful Encoding (Remembered > Forgotten)* | | | | | | | | | |
| none | -- | -- | -- | -- | -- | | -- | -- | |
| *Unsuccessful Encoding Forgotten > Remembered* | | | | | | | | | |
| Frontal |  |  |  |  |  | |  |  | |
| B Cingulate/Sup. Frontal G./Paracentral Lob. | B A23c, A24cd, A4ll, A6m, A8m | 2392 | -1 | -6 | 53 | | 6,8, 23-24 | ↓ | |
| Temporal |  |  |  |  |  | |  |  | |
| L Inf. Parietal Lob./Sup. Temporal G./  Post. Sup. Temporal S. | L A22c, A40c, A40rd, A40rv, A41/42, cpSTS, rpSTS | 3296 | -61 | -42 | 27 | | 22, 40-42 | ↓ | |
| Parietal |  |  |  |  |  | |  |  | |
| L Inf. Parietal Lob./Pre/Postcentral G. | L A1/2/3ulhf, A2, A40rd, A4hf, A4ul, A6cvl | 2344 | -54 | -17 | 48 | | 1-4, 6, 40 | ↓ | |
| R Inf. Parietal Lob./Postcentral G. | R A1/2/3ulhf, A2, A40rd | 720 | 57 | -20 | 50 | | 1-3, 40 | ↓ | |
| R Inf./Sup. Parietal/Pre/Postcentral G./  Paracentral Lob. | R A1/2/3ll, A1/2/3tru, A1/2/3ulhf, A2, A40rd, A4t, A4ul, A5l, A6cdl, A7ip, A7pc | 4536 | 30 | -30 | 64 | | 1-7, 40 | ↓ | |
| R Cingulate G./Precuneus/B Paracentral Lob. | B A1/2/3ll, A23c, A4ll, A5m | 1016 | 5 | -36 | 56 | | 1-3, 5, 23 | ↓ | |
| R Paracentral/Sup. Parietal Lob./Precuneus | R A1/2/3ll, A5m, A7c, A7ip, A7m, A7pc, A7r | 1112 | 20 | -54 | 57 | | 1-3, 5, 7 | ↓ | |
| Occipital |  |  |  |  |  | |  |  | |
| L Lat. Occipital Ctx. | L V5/MT+, mOccG | 648 | -38 | -80 | 6 | | 5 | ↓ | |
| Subcortical |  |  |  |  |  | |  |  | |
| B Basal Ganglia | R NAC, dCa, vCa | 640 | -2 | 12 | 6 | | - | ↓ | |
| *Note*. Significant clusters reflected only unsuccessful encoding of in-scanner foils (voxel-wise threshold of p < 0.02, cluster-wise threshold of p < 0.05). No successful encoding clusters were identified. Names in the brain region column are the anatomical labels associated with the Brainnetome atlas code. BA., Brodmann area; B, Bilateral; Ctx., Cortex; G., Gyrus; Inf., Inferior; L, Left; Lat., Lateral; Lob., Lobule; M.P., Monotonic pattern increasing (↑) or decreasing (↓)]; R, Right; S. Sulcus; Sup., Superior; Vol., Volume. | | | | | | | | |  |

| Supplementary Table 5. Brain regions where encoding-related vmPFC connectivity distinguished subsequently remembered from subsequently forgotten foils | | | | | | | | |  |
| --- | --- | --- | --- | --- | --- | --- | --- | --- | --- |
|  |  | Vol  (mm^3^) | MNI | | |  | |  | |
| Brain Region | Brainnetome Atlas Name |  | X | Y | Z | | B.A. | M.P. | |
| *Successful Encoding (Remembered > Forgotten)* | | | | | | | | | |
| Temporal/Parietal/Occipital |  |  |  |  |  | |  |  | |
| R Sup. Temporal G./Inf. Parietal Lob. | R A40rv, A41/42, TE1.01.2 | 584 | 45 | -30 | 20 | | 40-42 | ↑ | |
| L Sup. Temporal G./ Inf. Parietal Lob.  Post. Sup. Temporal S. | L A22c, A39rv, A40c, A40rv, A41/42, cpSTS, rpSTS | 832 | -49 | -46 | 24 | | 22, 39-42 | ↑ | |
| L Fusiform G./Inf. Temporal G./  Hippocampus/Post. Sup. Temporal S. | A37elv, A37lv, A37mv, A37vl, cHipp, cpSTS | 984 | -39 | -51 | -2 | | 37 | ↑ | |
| R Fusiform G./Inf. Temporal G./  Inf. Parietal Lob./ Lat. Occipital Ctx./  Post. Sup. Temporal S. | R A37lv, A37vl, A39c, V5/MT+, iOccG, RsOccG, mOccG, rpSTS | 1576 | 40 | -70 | 4 | | 5, 37, 39 | ↑ | |
| *Unsuccessful Encoding Forgotten > Remembered* | | | | | | | | | |
| Frontal |  |  |  |  |  | |  |  | |
| L Mid. Frontal/Cingulate G./  B. Sup. Frontal G. | B A10m, A9l, A9m, L A32p, A9/46d | 1496 | 0 | 56 | 28 | | 9-10, 32, 46 | ↓ | |
| R Mid./Sup. Frontal G. | R A46, A8vl, A9/46d, A9/46v, A9l | 2312 | 32 | 48 | 32 | | 8-9, 46 | ↓ | |
| R Inf./Mid. Frontal G. | R A45r, A46, A9/46v, IFS | 920 | 47 | 48 | 6 | | 9, 45-46 | ↓ | |
| R Inf./Orbital Frontal G. | R A11l, A12/47l, A12/47o, A45r | 648 | 47 | 44 | -15 | | 11-12, 45, 47 | ↓ | |
| Temporal |  |  |  |  |  | |  |  | |
| L Fusiform/Parahippocampal G./  MedioVentral Occipital Ctx./  Hippocampus | L A20rv, A37mv, TH, TL, cHipp, rLinG, vmPOS | 688 | -22 | -49 | -13 | | 20, 37 | ↓ | |
| Parietal |  |  |  |  |  | |  |  | |
| R Inf./Sup. Parietal Lob./  Pre/Postcentral G | R A1/2/3ulhf, A2, A40c, A40rd, A40rv, A4ul, A5l | 1960 | 50 | -24 | -13 | | 1-5, 40 | ↓ | |
| *Note*. Significant clusters reflected successful encoding or unsuccessful encoding of in-scanner foils (voxel-wise threshold of p < 0.02, cluster-wise threshold of p < 0.05). Names in the brain region column are the anatomical labels associated with the Brainnetome atlas code. BA., Brodmann area; B, Bilateral; Ctx., Cortex; G., Gyrus; Inf., Inferior; L, Left; Lat., Lateral; Lob., Lobule; M.P., Monotonic pattern increasing (↑) or decreasing (↓)]; R, Right; S. Sulcus; Sup., Superior; Vol., Volume. | | | | | | | | |  |
